# Analysis of docosahexaenoic acid hydroperoxide isomers in mackerel using liquid chromatography–mass spectrometry

**DOI:** 10.1038/s41598-023-28514-2

**Published:** 2023-01-24

**Authors:** Ibuki Kusumoto, Shunji Kato, Kiyotaka Nakagawa

**Affiliations:** Food Function Analysis Laboratory, Graduate School of Agricultural Science, Tohoku University, 468–1 Aramaki Aza Aoba, Aoba-ku, Sendai, Miyagi 980-8572 Japan

**Keywords:** Health care, Chemistry

## Abstract

Docosahexaenoic acid (DHA) is mostly esterified in food and is easily oxidized by exposure to heat or light. Hydroperoxide positions of DHA mono-hydroperoxide (DHA;OOH) provide information on oxidation mechanisms (e.g., radical- or singlet oxygen oxidation), yet direct identification of esterified DHA;OOH isomers has not been achieved. We previously accomplished the direct analysis of free DHA;OOH isomers with liquid chromatography-mass spectrometry (LC–MS/MS). In this study, we developed an LC–MS/MS method for direct analysis of esterified DHA;OOH based on our previous study. The developed method was capable of distinguishing esterified DHA;OOH isomers in raw- and oxidized mackerel. The result suggested that radical oxidation of esterified DHA can progress even in refrigeration. Different transitions were observed depending on the oxidation mechanism and lipid class. The analytical method and insights obtained in this study would be valuable to further understand and effectively prevent DHA oxidation in food products.

## Introduction

Docosahexaenoic acid (DHA) is an important fatty acid for both infants and adults since it is associated with brain development and functions^1–3^. Furthermore, studies have suggested that dietary intake of DHA can reduce the risk of neurodegenerative diseases and cardiovascular diseases^4–6^. However, DHA is readily oxidized during food processing, storage, or cooking, which compromises its nutritional value^7–9^. It has been suggested that excessive consumption of oxidized DHA may be associated with the development of various diseases, including cancer and cardiovascular disease^10–12^. Therefore, it is crucial to effectively prevent the oxidation of DHA in foods to maintain the nutritional value and physiological functions of DHA.

**Figure 1:**
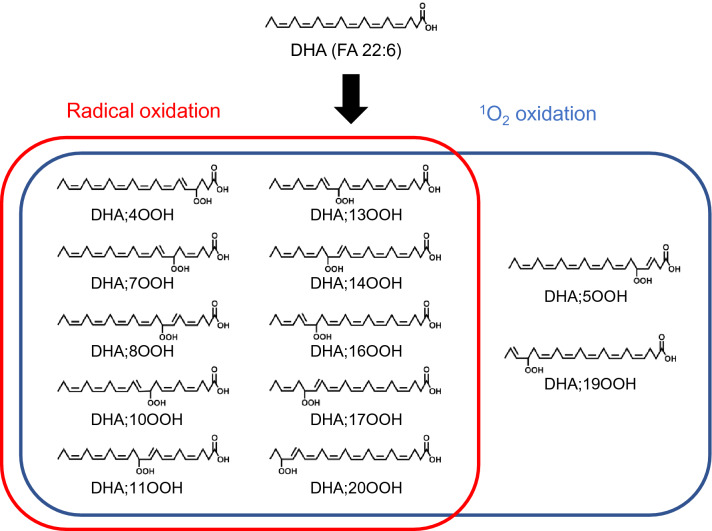
DHA;OOH isomers formed by radical- or ^1^O_2_ oxidation.

When DHA is oxidized, different isomers of DHA mono-hydroperoxide (DHA;OOH) are formed depending on the oxidation mechanisms (Fig. 1). In radical oxidation (e.g., thermal oxidation), DHA;OOH isomers whose hydroperoxyl groups are at carbons 4, 7, 8, 10, 11, 13, 14, 16, 17, or 20 are produced through the abstraction of hydrogen radical from a bis-allylic position of DHA. On the other hand, in addition to the isomers above, singlet oxygen (^1^O_2_) oxidation (e.g., Type II photo-oxidation) can yield DHA;5OOH and DHA;19OOH by direct reaction of ^1^O_2_ with double bonds. Thus, analyzing the position of hydroperoxyl groups of DHA;OOH isomers would help to identify the responsible oxidation mechanism. With this information, appropriate strategies could be implemented to prevent the oxidation of DHA (e.g., if thermal oxidation is known to be the most responsible oxidation mechanism for a specific food, tocopherol would be an effective antioxidant as it works as a radical scavenger.)

As mentioned above, DHA produces several hydroperoxide isomers (Fig. 1). DHA;OOH present in food is usually esterified to phospholipid phosphatidylcholine (PC-DHA) and triacylglycerol (TG-DHA). Hydroperoxide isomers of PC-DHA and TG-DHA (PC-DHA;OOH and TG-DHA;OOH) have generally been analyzed using gas chromatography after derivatization reactions, such as hydrogenation, methanolysis, and trimethylsilylation^13,14^. However, considering that DHA is easily oxidized, direct analysis of esterified DHA;OOH is preferable for stable analysis. To the best of our knowledge, there have not been any methods available for the direct discrimination of esterified DHA;OOH (e.g., PC-DHA;OOH and TG-DHA;OOH). Incidentally, Milne et al. introduced a method for the direct recognition of arachidic acid hydroperoxide esterified to PC with the characteristic Hock fragments in tandem mass spectrometry (MS/MS) analysis^15^, yet there remained a limitation in its selectivity. Under these circumstances, we recently developed a unique MS/MS method that can directly recognize hydroperoxide isomers of various lipids including free DHA;OOH by monitoring α-cleavage with high selectivity^16^. Thus, this method was considered useful for the accurate analysis of esterified DHA;OOH (e.g., PC-DHA;OOH and TG-DHA;OOH) isomers in various foods, including seafood products.

Given these circumstances, we focused on PC-DHA and TG-DHA species abundant in mackerel, which is widely consumed seafood rich in DHA. First, these hydroperoxides species were prepared through chemical reactions. With the prepared species, MS/MS methods were developed for the direct analysis of PC-DHA;OOH and TG-DHA;OOH. Their capability of discriminating hydroperoxyl group positions was then confirmed by analyzing raw and oxidized mackerel that underwent typical exposure to light or heat (e.g., storage and cooking). Different transitions were observed among the isomers in the oxidized mackerel based on the oxidation mechanisms. Thus, the developed methods provide a useful basis for preventing DHA oxidation in food products.

## Material and methods

### Materials

DHA and diolein (DG 18:1_18:1) were purchased from Nu-Check Prep, Inc (Elysian, MN, USA). Rose bengal, butyl hydroxytoluene (BHT), *N,N'*-dicyclohexylcarbodiimide (DCC), dimethylaminopyridine (DMAP), and 2-methoxypropene (MxP) were obtained from Wako Pure Chemical Industries, Ltd. (Osaka, Japan). Pyridinium *p*-toluenesulfonate (PPTS) was obtained from Sigma-Aldrich (St. Louis, MO, USA). 1-Palmitoyl-lysophosphatidylcholine (LPC 16:0/0:0) was obtained from Avanti Polar Lipids (Alabaster, AL, USA). All other reagents were of the highest grade available. Fresh raw mackerel fillet was purchased from a local grocery store in Sendai, Japan.

### Extraction of neutral and polar lipids in mackerel

Total lipid was extracted from raw mackerel (300 mg) using the modified Folch extraction method^17,18^. The obtained total lipid fraction was dissolved in 600 µL of chloroform/2-propanol (2:1, v/v). Total lipid (300 µL) was loaded onto an aminopropyl cartridge (Strata NH2, 55 µm, 70 Å, 100 mg, Phenomenex Inc. CA, USA), which was equilibrated with chloroform/2-propanol (2:1, v/v), and further eluted with chloroform/2-propanol (2:1, v/v, 2 mL). The eluent was collected as neutral lipids. Then, methanol (2 mL) was applied to elute phospholipids. The collected neutral lipids and phospholipids were dried and dissolved in 500 µL of hexane and 500 µL of methanol, respectively. These samples were stored at − 80 °C until the analysis. Prior to storage, the headspace of each tube was filled with N_2_ gas to prevent further oxidation.

### Analysis of the phospholipids extracted from mackerel

The extracted phospholipid fraction was diluted 1000-fold with methanol, and 1 µL of the solution was subjected to liquid chromatography (LC)-MS/MS consisting of ExionLC system (SCIEX, Tokyo, Japan) equipped with a 4000 QTRAP mass spectrometer (SCIEX). Separation was performed by isocratic elution with methanol/water (95:5, v/v) on a COSMOSIL Packed Column (5C_18_-MS-II, 5 μm, 2.0 ID × 250 mm, Nacalai Tesque Inc., Kyoto, Japan) at 45 °C. The flow rate was 0.2 mL/min. By mixing the column eluate with sodium acetate solution^19^, PC was detected as Na^+^ adduct in Q1 mass scan mode. Abundant PC molecular species were selected based on a Q1 mass scan, and their constituent fatty acids were determined by a product ion scan in direct infusion. The instruments were operated by Analyst software (ver. 1.7.2). The MS parameters are shown in Table S1 of Supplementary Material.

### Analysis of the neutral lipids extracted from mackerel

The extracted neutral lipid fraction was diluted 1000-fold with methanol, and 1 µL of the solution was injected into LC–MS/MS. Separation was performed by binary gradient elution with solvent A (methanol) and solvent B (2-propanol) on an ACQUITY UPLC HSS C18 Column (100 Å, 1.8 µm, 2.1 × 150 mm, Waters Corp, MA, USA) at 45 °C. The gradient program was as follows: 0–20 min, 20–100% B (0.2 mL/min). TG was detected as Na^+^ adducts in Q1 and neutral loss (NL) scan mode by mixing the column eluate with sodium acetate solution. TG molecular species in mackerel were analyzed by constructing 2-dimensional maps (2D maps)^20^. TG molecular species with the same total carbon number (C) are aligned on horizontal lines while those with the same total degree of unsaturation (n) are aligned on diagonal lines on 2D maps^21^. Based on these analyses, the spots consisting of TG-DHA molecular species were selected, and the other constituent fatty acids were further determined by product ion scan. The MS parameters are shown in Table S1 of Supplementary Material.

### Model sample preparation of a mixture of PC-DHA;OOH isomers

PC 16:0/22:6 was determined as a target since it was abundant in mackerel based on the analysis of the extracted phospholipid fraction. A mixture of its hydroperoxide isomers (i.e., PC 16:0/22:6;OOH isomers) was prepared as a model sample of PC-DHA;OOH.

DHA (399 mg) was esterified to LPC 16:0/0:0 (200 mg) in chloroform (26.9 mL) containing DCC (812 mg) and DMAP (234 mg) under N_2_ gas for 28 h at 40 °C^22^. After the reaction, the phospholipid fraction was collected with Sep-Pak Vac Silica (35 cc, 10 g, Waters, MA, USA)^17^. The PC 16:0/22:6 was chromatographically purified as in our previous report^23^. PC 16:0/22:6 was further purified by removing the salt contained in the collected fraction. The purified PC 16:0/22:6 was dissolved into methanol (20 mL).

Rose bengal (0.15 mg) was added to the PC solution, and photo-oxidation was carried out by exposing the solution to LED irradiation (6000 lx, on ice) for 3 h. Rose bengal was removed with Sep-Pak Accell Plus QMA (360 mg, Waters). The eluent was dried under N_2_ gas. PC 16:0/22:6;OOH was purified in the same manner as the PC 16:0/22:6 purification. The total weight of obtained PC 16:0/22:6;OOH isomers was 1.6 mg after the collected eluent was dried. The sample was dissolved in chloroform (200 μL) and stored at − 80 °C until the analysis. The headspace of the sample vial was filled with N_2_ gas prior to storage.

### Model sample preparation of a mixture of TG-DHA;OOH isomers

TG 18:1_18:1_22:6 was determined as a target since it was abundant in mackerel based on the analysis of the extracted neutral lipid fraction. A mixture of its hydroperoxide isomers (i.e., TG 18:1_18:1_22:6;OOH isomers) was prepared as a model sample of TG-DHA;OOH.

While PC 16:0/22:6;OOH was prepared by photo-oxidation of PC 16:0/22:6, TG 18:1_18:1_22:6 being exposed to LED irradiation would generate TG 18:1_18:1;OOH 22:6 in addition to TG 18:1_18:1_22:6;OOH. Thus, the desired product (i.e., TG 18:1_18:1_22:6;OOH) was exclusively prepared by esterification of DHA;OOH (protected with MxP) to DG 18:1_18:1.

DHA (1 g) was dissolved in 24 mL of methanol containing rose bengal (0.52 mg). The solution was exposed to LED irradiation (6000 lx, 4 °C) for approximately 17 h. Rose bengal was removed by the same procedure as in 2.5. The mixture of DHA;OOH isomers and unoxidized DHA were collected by a semi-preparative LC^19^. The exposure of unoxidized DHA to LED irradiation was repeated to further collect DHA;OOH. The hydroperoxyl group of the collected DHA;OOH was protected with MxP and esterified to DG 18:1_18:1 as in previous studies^19,22^. After deprotection, the resultant mixture of TG 18:1_18:1_22:6;OOH isomers weighed 9.2 mg. The sample was stored at − 80 °C until the analysis. The headspace of the sample vial was filled with N_2_ gas prior to storage.

### Photo- and thermal oxidation of mackerel

A raw mackerel fillet was cut into single pieces that weigh around 300 mg each. The fillet did not contain a head or fins. The skin was removed before cutting. For photo-oxidation, a piece of the cut mackerel was put into a 10 mL clear glass tube. Each tube was capped and exposed to LED light in a cool incubator (2000 lx, 4 °C) for 24, 48, or 72 h. For thermal oxidation, a piece of the cut mackerel was put into a 10 mL amber glass tube. Each tube was heated with its caps on at 100 °C on a block heater (TB-620 Hot Block Baths, Advantec Co., Ltd., Tokyo, Japan) for 4, 6, or 8 min. Each sample was flipped once in a tube so that the heat transferred evenly. Phospholipids and neutral lipids were extracted from 50 mg of photo- and thermally oxidized mackerel in the same manner as in 2.3, and reconstructed in 500 μL of methanol and hexane, respectively. The extracted samples were stored at − 80 °C until analysis.

### PC-DHA;OOH and TG-DHA;OOH isomers analyses in the prepared model samples and mackerel extract by LC–MS/MS

To develop the direct analysis method of PC-DHA;OOH and TG-DHA;OOH isomers, each model sample of PC 16:0/22:6;OOH isomers mixture and TG 18:1_18:1_22:6;OOH isomers mixture was infused into 6500 QTRAP mass spectrometer (SCIEX), to obtain Q1 mass and product ion mass spectra. To distinguish the hydroperoxide position of each isomer, their specific product ions were selected. Using selected product ions, multiple reaction monitoring (MRM) pairs were constructed and the MS parameters were optimized (Table S2 and Table S3 of Supplementary Material). The instruments were operated by Analyst software (ver. 1.7.2.).

Using constructed MRM pairs, LC–MS/MS conditions for PC 16:0/22:6;OOH and TG 18:1_18:1_22:6;OOH isomers were optimized with the ExionLC system (SCIEX) equipped with a 6500 QTRAP. Separation of PC 16:0/22:6;OOH was performed by binary gradient elution with solvent A (water) and solvent B (methanol) on a COSMOSIL Packed Column (5C_18_-MS-II, 5 μm, 2.0 ID × 250 mm, Nacalai Tesque Inc.) at 40 °C. The gradient program was as follows: 0–15 min, 90–97.5% B linear at 0.2 mL/min. The column eluent was mixed with a post-column solvent consisting of methanol containing 2 mM of sodium acetate at 0.01 mL/min to promote ionization. For TG 18:1_18:1_22:6;OOH isomers, the separation was carried out by binary gradient elution consisting of solvent A (hexane/2-propanol, 100:1, v/v) and solvent B (hexane/2-propanol, 1000:5, v/v) on an Intertsil SIL-100A (3 μm, 2.1 × 250 mm, GL Sciences Inc., Tokyo, Japan) at 40 °C. The gradient program was as follows: 0–26 min, 0–86.7% A linear at 0.2 mL/min. The column eluent was mixed with a post-column solvent consisting of methanol/2-propanol (4:1, v/v) containing 0.2 mM of sodium acetate at 0.1 mL/min to promote ionization.

A portion (10 μL) of each mackerel sample obtained in 2.7 was analyzed using the optimized LC–MS/MS conditions.

## Results and discussion

### Determination of target PC and TG molecular species with LC–MS/MS for the analyses of esterified DHA;OOH

As mentioned in the introduction, DHA is primarily present in esterified forms (e.g., PC-DHA and TG-DHA) in food products, composing a variety of molecular species with different combinations of the other constituent fatty acid/acids. Thus, for the later analysis of esterified DHA;OOH isomers, suitable targets of PC-DHA and TG-DHA molecular species that are abundant in mackerel were first determined with LC–MS/MS.

**Figure 2:**
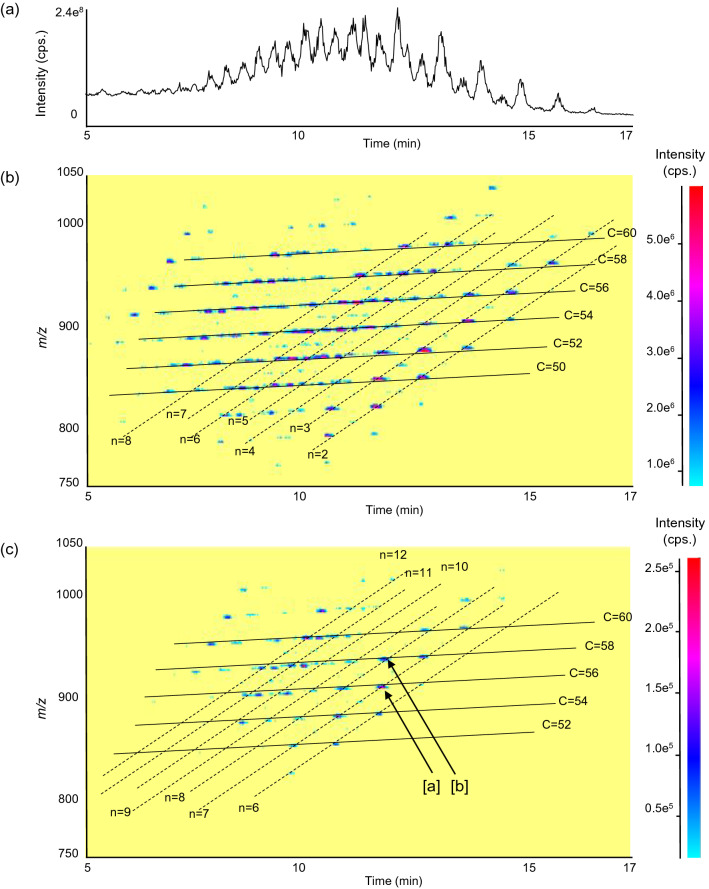
(**a**) LC–MS chromatogram of neutral lipid fraction extracted from mackerel. (**b**) 2D map of Q1 scan of neutral lipid fraction extracted from mackerel. (**c**) 2D map of NL scan of neutral lipid fraction extracted from mackerel for the NL of 328.2 Da. The 2D maps in this figure were generated by using Analyst software (ver. 1.6.3) under AB SCIEX Software License.

The phospholipid fraction extracted from mackerel was analyzed by LC–MS/MS. As described in Section “Analysis of the phospholipids extracted from mackerel”, all ions were detected as Na^+^ adducts to simplify the discrimination of precursor ions, and five major peaks were observed on the chromatogram (Figure S1a of Supplementary Material). The highest peak was mainly detected at *m/z* 828.9, and a product ion scan of *m/z* 828.9 was then performed for further structural analysis (Figure S1b of Supplementary Material). As a result, neutral losses specific to PC (i.e., 59.1 Da (trimethylamine) and 183.1 Da (phosphocholine)) were observed, thus ensuring that the peak of *m/z* 828.9 was PC. Additional peaks of *m/z* 513.4 and *m/z* 441.7 were expected to be produced by the dissociation of trimethylamine (59.1 Da) along with FA 16:0 (256.2 Da) and DHA (328.2 Da), respectively^24^. The *sn*-positional distribution was determined based on the intensity of *m/z* 513.4 and *m/z* 441.7^24^, and *m/z* 828.9 in the largest peak was identified as PC 16:0/22:6, which was selected as the target PC. (PC species in other lower peaks are discussed in Figure S1a of Supplementary Material.) Unlike the phospholipid fraction, the chromatogram of LC–MS/MS analysis of the neutral lipid fraction suggested the vast diversity of TG molecular species in mackerel showing a great number of peaks (Fig. 2a). Thus, a comprehensive TG analysis with 2D maps was further performed. Ikeda et al. successfully conducted an analysis of TG molecular species in lipid mixtures of biological samples (mouse liver and white adipose tissue) by 2D maps drawn with retention time (x-axes) and *m/z* (y-axes) of LC–MS analysis^21^. Our group further applied 2D maps for the analysis of vegetable oils^20^. In this study, we confirmed the applicability of 2D maps for food products (mackerel) as a plethora of TG molecular species were systematically aligned on its map (Fig. 2b). To exclusively analyze TG molecular species that possess DHA (monoisotopic mass: 328.240 Da), a 2D map of neutral loss scan for 328.2 Da was constructed. Only diagonal lines of n ≥ 6 appeared on the 2D map as DHA bears six double bonds (Fig. 2c). Product ion scan was then conducted for *m/z* of spots with high intensity. For instance, spot [a] (C = 56, n = 7, *m/z* 927.9) showed product ions derived by the loss of DHA, FA 14:0, FA 16:0, FA 18:1, FA 20:5, and FA 20:1 (Figure S2 of Supplementary Material). Because the spot [a] was on the line of C = 56 and n = 7, TG 16:0_18:1_22:6, TG 14:0_20:1_22:6, and TG 18:1_18:1_20:5 were expected to be contained in the spot. Meanwhile, spot [b] (*m/z* 953.7, C = 58, n = 8) mainly showed product ions corresponding to DHA and FA 18:1. In this case, only TG 18:1_18:1_22:6 satisfied the condition (i.e., C = 58 and n = 8). Targeting *m/z* of the spot [b] would make TG-DHA;OOH analysis simpler in contrast to the spot [a] which contains multiple TG molecular species. Therefore, we considered that TG 18:1_18:1_22:6 was a suitable target that bears DHA.

### MS/MS and LC–MS/MS analyses of PC-DHA;OOH and TG-DHA;OOH in the prepared model samples

As described in the introduction, we have previously developed an MS/MS method that can directly identify isomers of lipid hydroperoxides by monitoring the characteristic product ions obtained by fragmentation near the hydroperoxyl group in the presence of Na^+^^16,17,19^. With the use of this method, for instance, we were able to discriminate the twelve isomers of free DHA;OOH (Fig. 1), and we expected it to also be applicable for analyzing the more complex esterified DHA;OOH (e.g., PC-DHA;OOH and TG-DHA;OOH). In the previous section, PC 16:0/22:6 and TG 18:1_18:1_22:6 were determined as target species that possess DHA in mackerel. Here, prior to the analysis of the mackerel sample, hydroperoxides of these targets were prepared through photo-oxidation and used as model samples to evaluate whether the hydroperoxyl group positions of their isomers could be determined by our MS/MS method.

**Figure 3:**
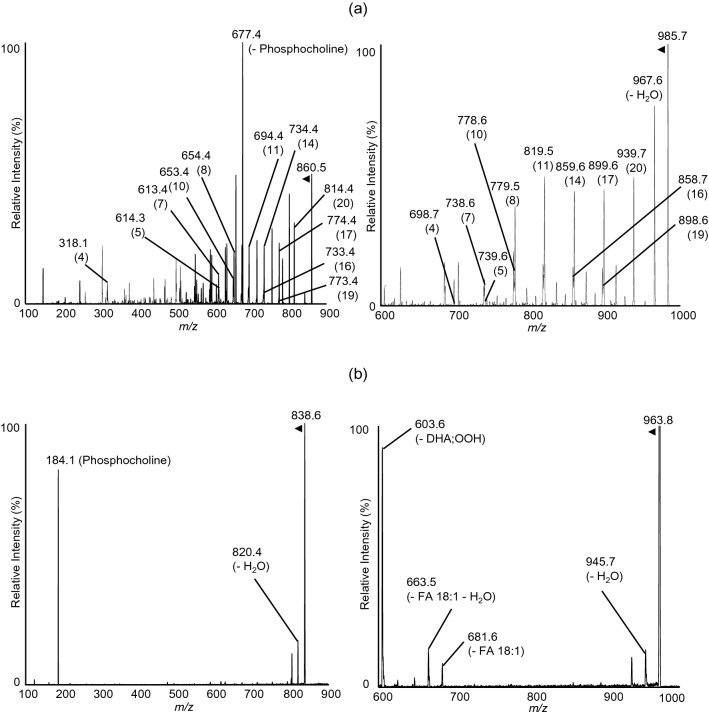
(**a**) Product ion scan of Na^+^ adduct of PC 16:0/22:6 (left) and TG 18:1_18:1_22:6 (right) in the model samples. (**b**) Product ion scan of H^+^ adduct of PC 16:0/22:6 (left) and TG 18:1_18:1_22:6 (right) in the model samples. Na^+^ adduct of PC 16:0/22:6;OOH showed product ions derived by the dissociation of phosphocholine or trimethylamine with the isomer-specific neutral losses.

The model sample of PC 16:0/22:6;OOH was obtained through photo-oxidation of the synthesized PC 16:0/22:6. Since oxidation of TG 18:1_18:1_22:6 could produce TG 18:1_18:1;OOH_22:6 as well as the targeted TG;OOH (i.e., TG 18:1_18:1 _22:6;OOH), TG 18:1_18:1_22:6;OOH was prepared by deprotection of MxP-protected DHA;OOH (DHA;OO-MxP) esterified into DG 18:1_18:1. The Q1 mass spectra of the model samples showed *m/z* 860.4 and *m/z* 985.7, which correspond to Na^+^ adduct of PC 16:0/22:6;OOH and TG 18:1_18:1_22:6;OOH, respectively (data not shown). Each of the product ion spectrum for the detected Na^+^ adduct of these hydroperoxides showed all of the twelve product ions that were expected according to the fragmentation rule discovered in our previous studies^16,17,19^ (Fig. 3a). In contrast, the product ion spectrum of H^+^ adducts of PC 16:0/22:6;OOH and TG 18:1_18:1_22:6;OOH did not show any product ions that provide information on hydroperoxide group positions (Fig. 3b). Thus, we confirmed the occurrence of the position-specific fragmentation in Na^+^ adducts of esterified DHA;OOH (i.e., PC-DHA;OOH and TG-DHA;OOH), which enabled direct detection of their hydroperoxide positions without any derivatizations.

**Figure 4:**
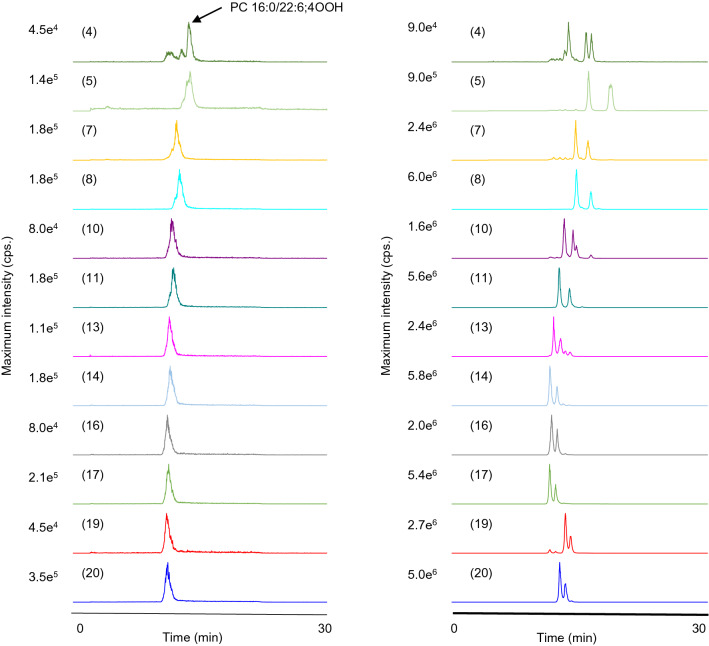
LC–MS/MS chromatograms of the model samples of PC 16:0/22:6 (left) and TG 18:1_18:1_22:6 (right). Values given in parentheses indicate the hydroperoxyl group position. The chromatogram of PC 16:0/22:6;4OOH showed additional lower peaks. Considering the collision energy set for PC 16:0/22:6;4OOH was relatively high (44 eV), these additional peaks were possibly derived by further dissociation of the other isomers. All isomers of TG 18:1_18:1_22:6;OOH were distinctly observed on their chromatograms. The combination of silica-based normal phase and hexane was applied to the analysis for TG 18:1_18:1_22:6;OOH isomers as normal phase LC is known to be effective to recognize hydroperoxyl group position. In normal-phase LC, *cis*-, *trans*-isomers and *sn*-positional isomers of TG hydroperoxides can be separated. In this study, DHA;OOH was prepared by photo-oxidation with white LED irradiation, thus it can be presumed that conversion from *cis*-isomers to *trans*-isomers did not occur in the process of the TG 18:1_18:1_22:6;OOH preparation. Therefore, TG 18:1_18:1(*sn-2*)_22:6;OOH and TG 18:1_22:6;OOH(*sn-2*)_18:1 were expected to account for the observed peaks on each chromatogram. In the case of TG 18:1_18:1_22:6;4OOH, three peaks are observed on its chromatogram. Considering TG 18:1_18:1(*sn-2*)_22:6;OOH was prepared from achiral materials, the resulting reference was expected to be a racemic mixture with the ratio of the two pairs of diastereomers being 50%. With that respect, the latter two peaks on the chromatogram of TG 18:1_18:1_22:6;4OOH presumably consisted of diastereomers. Likewise, the semi-separated peaks of TG 18:1_18:1_22:6;5OOH seemed to be explained by the separation of TG 18:1_18:1(*sn-2*)_22:6;5OOH diastereomers.

Based on the observed product ions, we were able to set up MRM pairs for every isomer of PC 16:0/22:6;OOH and TG 18:1_18:1_22:6;OOH. MS/MS was then connected to LC, and the LC–MS/MS conditions were optimized for the selected MRM pairs to analyze each of the model samples. In general, reversed-phase or normal-phase columns are used in LC–MS/MS analysis for PC;OOH and TG;OOH isomers^25,26^; nevertheless, it is difficult to analyze the isomers with high accuracy since they cannot be completely separated from each other in LC^27–30^. In the present study, while the isomers of PC 16:0/22:6;OOH and TG 18:1_18:1_22:6;OOH were not fully separated by LC as in the previous studies (Fig. 4), the highly selective MRM made it possible to accurately analyze each isomer. As a side note, the following characteristics were observed on the MRM chromatograms. Most of the chromatograms of PC 16:0/22:6;OOH isomers clearly showed a single peak, yet that of PC 16:0/22:6;4OOH had additional peaks besides the peak of the isomer, which might be attributed to the relatively higher collision energy set for the MRM pair (44 eV). The normal-phase LC condition was seemingly able to separate their DHA;OOH-positional isomers on the glycerol backbone and/or diastereomeric structure^19,31–33^, which is described in detail in Fig. 4.

In this regard, the selected MRM was efficient for each isomer of both PC 16:0/22:6;OOH and TG 18:1_18:1_22:6;OOH, thus overcoming limitations in indirect analysis methods for hydroperoxide isomers^13–15^. On top of that, oxidized and non-oxidized lipids (e.g., PCOOH and PC) are sufficiently separated under the present conditions (data not shown), thus we considered that the sample (mackerel) can be analyzed without much influence from matrices or ion suppression.

### Analysis of photo- or thermally oxidized mackerel

In the previous section, it was confirmed that hydroperoxyl group positions of esterified DHA;OOH could be determined with our LC–MS/MS method. Accordingly, raw and oxidized (photo-irradiated or heated) mackerel were analyzed in the same manner. Raw mackerel was oxidized by either exposure to light (4 °C, 2000 lx) or heat (100 °C, in shade), assuming storage or cooking conditions in daily consumption.

**Figure 5:**
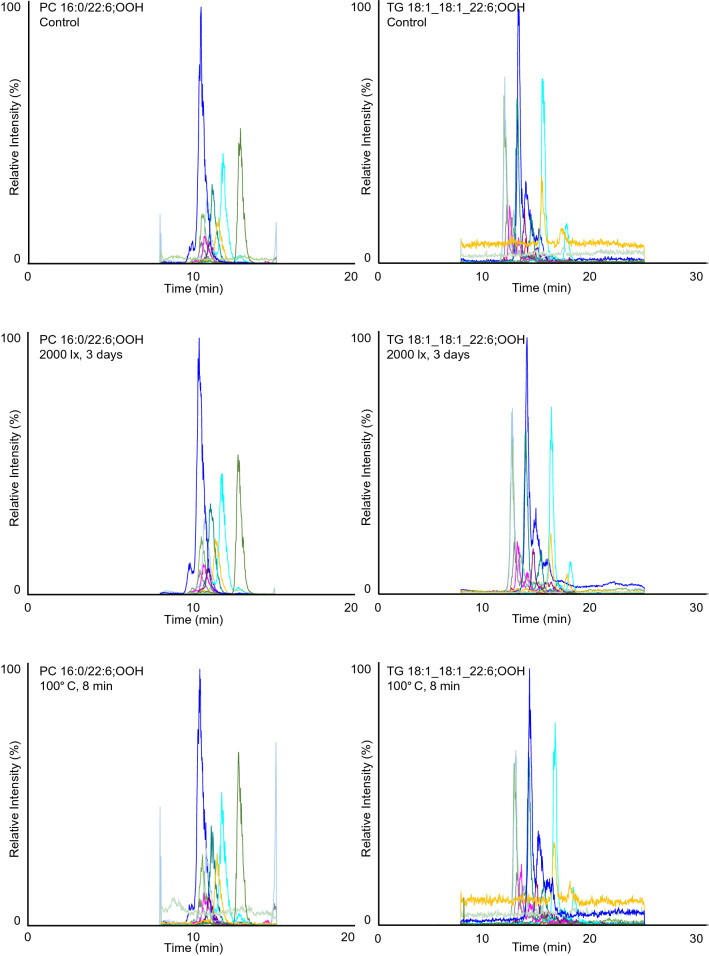
Typical LC–MS/MS chromatograms of PC 16:0/22:6;OOH (left) and TG 18:1_18:1_22:6;OOH (right) in mackerel.

**Figure 6:**
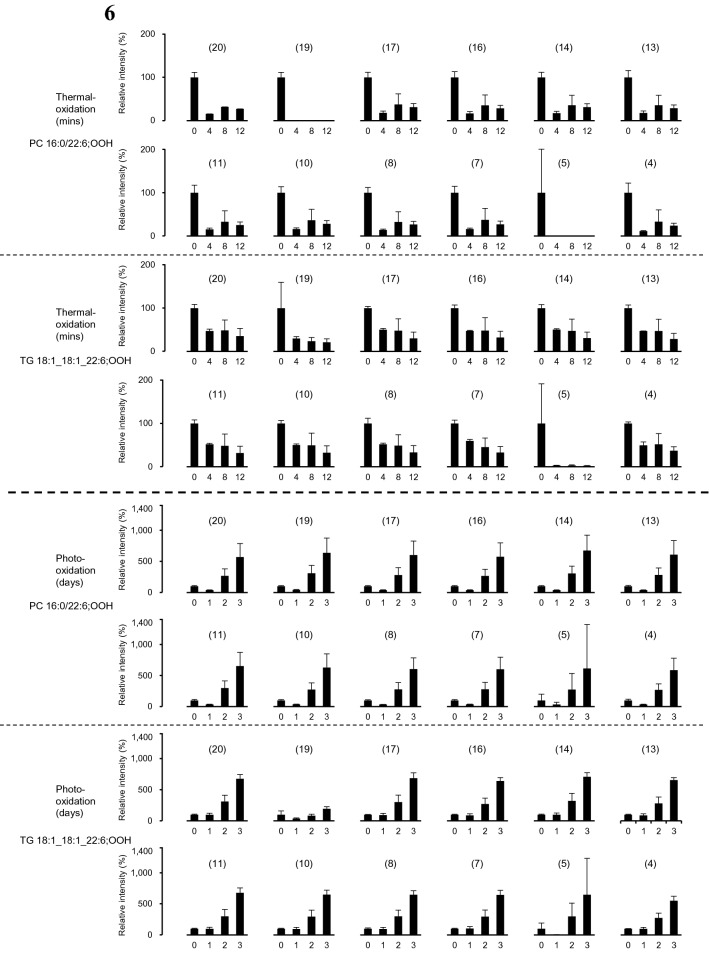
Relative peak area of PC 16:0/22:6;OOH isomers and TG 18:1_18:1_22:6;OOH isomers in mackerel. Values given in parentheses indicate the hydroperoxyl group position. Values are expressed as mean ± SD, n = 3.

The typical chromatograms of photo- or thermally oxidized mackerel are shown in Fig. 5 and Figure S3 of Supplementary Material. The target PC 16:0/22:6;OOH and TG 18:1_18:1_22:6;OOH were distinctly detected even in fresh mackerel, which corroborated the high sensitivity and selectivity of the constructed MRM (Figure S3a of Supplementary Material). In the LED-irradiated mackerel, the occurrence of ^1^O_2_ oxidation was confirmed by the increase of the isomers uniquely produced by ^1^O_2_ oxidation (i.e., PC 16:0/22:6;5OOH, PC 16:0/22:6;19OOH, TG 18:1_18:1_22:6;5OOH, and TG 18:1_18:1_22:6;19OOH), thus indicating that some photosensitizers were present in raw mackerel (Fig. 6). To the best of our knowledge, this is the first report that detected ^1^O_2_ oxidation-specific DHA;OOH isomers in seafood products. The other isomers of target PC-DHA;OOH and TG-DHA;OOH increased in mackerel as well (Fig. 6). Interestingly, the increase rates of the ^1^O_2_ oxidation-specific isomers were overall lower than that of the other isomers, which indicated that radical oxidation can also progress in esterified DHA even at a low temperature (4 °C). In thermal-oxidation (i.e., radical oxidation), all isomers of both PC 16:0/22:6;OOH and TG 18:1_18:1_22:6;OOH tended to decrease throughout the oxidation period corroborating the instability of DHA;OOH^34^ (Fig. 6). Particularly, the target PC-DHA;OOH and TG-DHA;OOH with their hydroperoxyl groups at carbon 5 or 19 rapidly decreased. As described in the introduction, DHA;5OOH and DHA;19OOH are not produced by radical oxidation. Thus, the decrease of the target PC-DHA;OOH and TG-DHA;OOH isomers were thought to be reflecting their decomposition. Additionally, the reduction rate was somewhat higher in PC 16:0/22:6;OOH isomers than TG 18:1_18:1_22:6;OOH isomers, which suggested that PC;OOH is more susceptible to thermal breakdown than TG;OOH. It would also be possible that PC is more resistant to thermal oxidation than TG, and thus PC 16:0/22:6;OOH present in raw mackerel rapidly decreased during heating^35–37^. In contrast to the difference between the breakdown of PC 16:0/22:6;OOH and TG 18:1_18:1_22:6;OOH during heating, the photo-oxidized mackerel did not show any considerable differences between productions of PC 16:0/22:6;OOH and TG 18:1_18:1_22:6;OOH.

## Conclusion

In most of the previous studies, hydroperoxide positional isomers of esterified DHA were analyzed with gas chromatography (GC)-MS, which entails apprehension of negative effects on its accuracy due to artifacts that could be produced during derivatization (e.g., hydrogenation, methanolysis, trimethylsilylation). As described in the introduction, an LC–MS/MS analysis for esterified hydroperoxide was previously introduced, but the complete discrimination of their isomers still remained to be desired. Our MS/MS analysis accomplished both direct and selective detection of all the twelve isomers of esterified DHA;OOH in food samples based on the fragmentation rule discovered in our previous studies. It is worth mentioning that DHA;5OOH and DHA;19OOH, which are specifically formed by ^1^O_2_ oxidation, were successfully analyzed for the first time. The increasing tendency of these isomers in LED-irradiated mackerel suggested that radical oxidation can progress even at a low temperature. Meanwhile, all isomers were decreasing in mackerel during heating likely due to their rapid decomposition. Furthermore, the decreased rate of the targeted PC-DHA;OOH was overall higher than that of the targeted TG-DHA;OOH in heated mackerel, indicating different stability to thermal oxidation among different lipid classes. By this means, our LC–MS/MS analysis provides valuable information on oxidation mechanisms that DHA in food products goes through. The analysis introduced in this paper would contribute to developing more optimized prevention of DHA oxidation in various food products. Further studies are necessary to elucidate the mechanisms which caused the difference observed between the production of DHA;OOH isomers in different lipid classes (i.e., PC and TG). More replication of our findings in a diverse range of food products (e.g., other types of fish rich in DHA including salmon and sardine, shellfish, algae oil, or their processed food products and supplements) with a larger sample size would strengthen the insight and provide a better understanding of DHA oxidation occurring in food. Moreover, this analysis could be utilized to investigate the potential impact of DHA oxidation on human health. Besides possible adverse effects of DHA hydroperoxides, their involvement in metabolism or cell function could also be explored as recent studies have revealed the biological functions of DHA-derived oxylipins^38^.

## Supplementary Information

Supplementary Information.

## Data Availability

The datasets used and/or analyzed during this study are available from the corresponding author upon reasonable request.

## Abbreviations

DHA: Docosahexaenoic acid
PC-DHA: Phosphatidylcholine that possesses DHA
TG-DHA: Triacylglycerol that possesses DHA
DHA;OOH: DHA mono-hydroperoxide
^1^O_2_ oxidation: Singlet oxygen oxidation
DHA;xOOH: DHA;OOH whose hydroperoxyl group is placed at carbon number x
PC-DHA;OOH: PC-DHA hydroperoxide with DHA;OOH
TG-DHA;OOH: TG-DHA hydroperoxide with DHA;OOH
MS/MS: Tandem mass spectrometry
2D maps: 2-dimensional maps
DG 18:1_18:1: Diolein
BHT: Butyl hydroxytoluene
DCC: *N,N'*-Dicyclohexylcarbodiimide
DMAP: Dimethylaminopyridine
MxP: 2-Methoxypropene
PPTS: Pyridinium *p*-toluenesulfonate
LPC 16:0/0:0: 1-Palmitoyl-lysophosphatidylcholine
HPLC: High-performance liquid chromatography
LC–MS: HPLC-mass spectrometry
LC–MS/MS: HPLC-tandem mass spectrometry
MRM: Multiple reaction monitoring
NL: Neutral loss
