# Supplementary material for: Analysis of docosahexaenoic acid hydroperoxide isomers in mackerel using liquid chromatography–mass spectrometry
Source: Sci Rep. 2023 Jan 24;13:1325. doi: 10.1038/s41598-023-28514-2 (PMC9873796; doi:10.1038/s41598-023-28514-2)
Supplement: Supplementary file 1 — Supplementary Information. [file 41598_2023_28514_MOESM1_ESM.pdf]

Supplementary Material

Analysis of docosahexaenoic acid hydroperoxide isomers in mackerel using liquid chromatography-mass spectrometry

Ibuki KUSUMOTO[1], Shunji KATO[1], Kiyotaka NAKAGAWA[1*]

[1]*Food Function Analysis Laboratory, Graduate School of Agricultural Science, Tohoku University, Sendai, Miyagi 980-8572, Japan*

[*]**Corresponding author:** Food Function Analysis Laboratory, Graduate School of Agricultural Science, Tohoku University, 468–1 Aramaki Aza Aoba, Aoba-ku, Sendai, 980-8572, Japan. Fax: +81-22-757-4417.

E-mail address: kiyotaka.nakagawa.c1@tohoku.ac.jp

Table S1

	PC		TG			
	Q1	MS2	Q1	NL	MS2<br>TG 16:0_18:1_22:6	MS2<br>TG 18:1_18:1_22:6
Source	ESI		ESI			
Ion polarity	Positive		Positive			
Declustering potential (V)	125	125	125	125	125	130
Entrance potential (V)	10	10	10	10	10	10
Temperature (°C)	650	300	650	650	650	600
Ion spray voltage (V)	5500	5500	5500	5500	5500	5500
Curtain gas (psi)	20	20	20	20	20	30
Collision-activated dissociation gas (psi)	-	4	-	4	4	9
Collision energy (V)	-	55	-	51	51	50
Collision cell exit potential (V)	-	10	-	14	14	10
Ion source gas 1 (psi)	50	50	50	50	50	40
Ion source gas 2 (psi)	60	80	60	60	60	60

Table S1 MS parameters used for the analysis of PC and TG in mackerel.
Analysis was conducted with a 4000 QTRAP mass spectrometer (SCIEX) except for TG 18:1_18:1_22:6.
TG 18:1_18:1_22:6 was analyzed with a 6500 QTRAP mass spectrometer (SCIEX).

Table S2

	PC 16:0/22:6;20OOH	PC 16:0/22:6;19OOH	PC 16:0/22:6;17OOH	PC 16:0/22:6;16OOH
Source		ESI		
Ion polarity		Positive		
Declustering potential (V)		200		
Entrance potential (V)		10		
Temperature (°C)		500		
Ion spray voltage (V)		5500		
Curtain gas (psi)		30		
Collision-activated dissociation gas (psi)		9		
Collision energy (V)	36	35	35	34
Collision cell exit potential (V)	15	15	15	20
Ion source gas 1 (psi)		40		
Ion source gas 2 (psi)		60		
Precursor ion (*m/z*)		860.4		
Product ion (*m/z*)	814.4	773.5	774.4	733.4

	PC 16:0/22:6;14OOH	PC 16:0/22:6;13OOH	PC 16:0/22:6;11OOH	PC 16:0/22:6;10OOH
Source		ESI		
Ion polarity		Positive		
Declustering potential (V)		200		
Entrance potential (V)		10		
Temperature (°C)		500		
Ion spray voltage (V)		5500		
Curtain gas (psi)		30		
Collision-activated dissociation gas (psi)		9		
Collision energy (V)	37	35	36	35
Collision cell exit potential (V)	15	20	15	20
Ion source gas 1 (psi)		40		
Ion source gas 2 (psi)		60		
Precursor ion (*m/z*)		860.4		
Product ion (*m/z*)	734.4	693.3	694.4	653.5

	PC 16:0/22:6;8OOH	PC 16:0/22:6;7OOH	PC 16:0/22:6;5OOH	PC 16:0/22:6;4OOH
Source		ESI		
Ion polarity		Positive		
Declustering potential (V)		200		
Entrance potential (V)		10		
Temperature (°C)		500		
Ion spray voltage (V)		5500		
Curtain gas (psi)		30		
Collision-activated dissociation gas (psi)		9		
Collision energy (V)	38	36	42	44
Collision cell exit potential (V)	22	22	24	15
Ion source gas 1 (psi)		40		
Ion source gas 2 (psi)		60		
Precursor ion (*m/z*)		860.4		
Product ion (*m/z*)	654.4	613.4	614.3	318.1

Table S2 MS parameters used for the analysis of PC 16:0/22:6;OOH.

Table S3

	TG 18:1_18:1_22:6;20OOH	TG 18:1_18:1_22:6;19OOH	TG 18:1_18:1_22:6;17OOH	TG 18:1_18:1_22:6;16OOH
Source	ESI			
Ion polarity	Positive			
Declustering potential (V)	210			
Entrance potential (V)	10			
Temperature (°C)	500			
Ion spray voltage (V)	5500			
Curtain gas (psi)	30			
Collision-activated dissociation gas (psi)	9			
Collision energy (V)	45	37	44	35
Collision cell exit potential (V)	20	25	25	30
Ion source gas 1 (psi)	40			
Ion source gas 2 (psi)	60			
Precursor ion (*m/z*)	985.7			
Product ion (*m/z*)	939.6	898.6	899.6	858.7

	TG 18:1_18:1_22:6;14OOH	TG 18:1_18:1_22:6;13OOH	TG 18:1_18:1_22:6;11OOH	TG 18:1_18:1_22:6;10OOH
Source	ESI			
Ion polarity	Positive			
Declustering potential (V)	210			
Entrance potential (V)	10			
Temperature (°C)	500			
Ion spray voltage (V)	5500			
Curtain gas (psi)	30			
Collision-activated dissociation gas (psi)	9			
Collision energy (V)	45	36	38	35
Collision cell exit potential (V)	25	28	30	30
Ion source gas 1 (psi)	40			
Ion source gas 2 (psi)	60			
Precursor ion (*m/z*)	985.7			
Product ion (*m/z*)	859.6	818.6	819.6	778.6

	TG 18:1_18:1_22:6;8OOH	TG 18:1_18:1_22:6;7OOH	TG 18:1_18:1_22:6;5OOH	TG 18:1_18:1_22:6;4OOH
Source	ESI			
Ion polarity	Positive			
Declustering potential (V)	210			
Entrance potential (V)	10			
Temperature (°C)	500			
Ion spray voltage (V)	5500			
Curtain gas (psi)	30			
Collision-activated dissociation gas (psi)	9			
Collision energy (V)	44	36	46	44
Collision cell exit potential (V)	25	30	35	30
Ion source gas 1 (psi)	40			
Ion source gas 2 (psi)	60			
Precursor ion (*m/z*)	985.7			
Product ion (*m/z*)	779.5	738.6	739.6	698.7

Table S3 MS parameters used for the analysis of TG 18:1_18:1_22:6;OOH

Figure S1

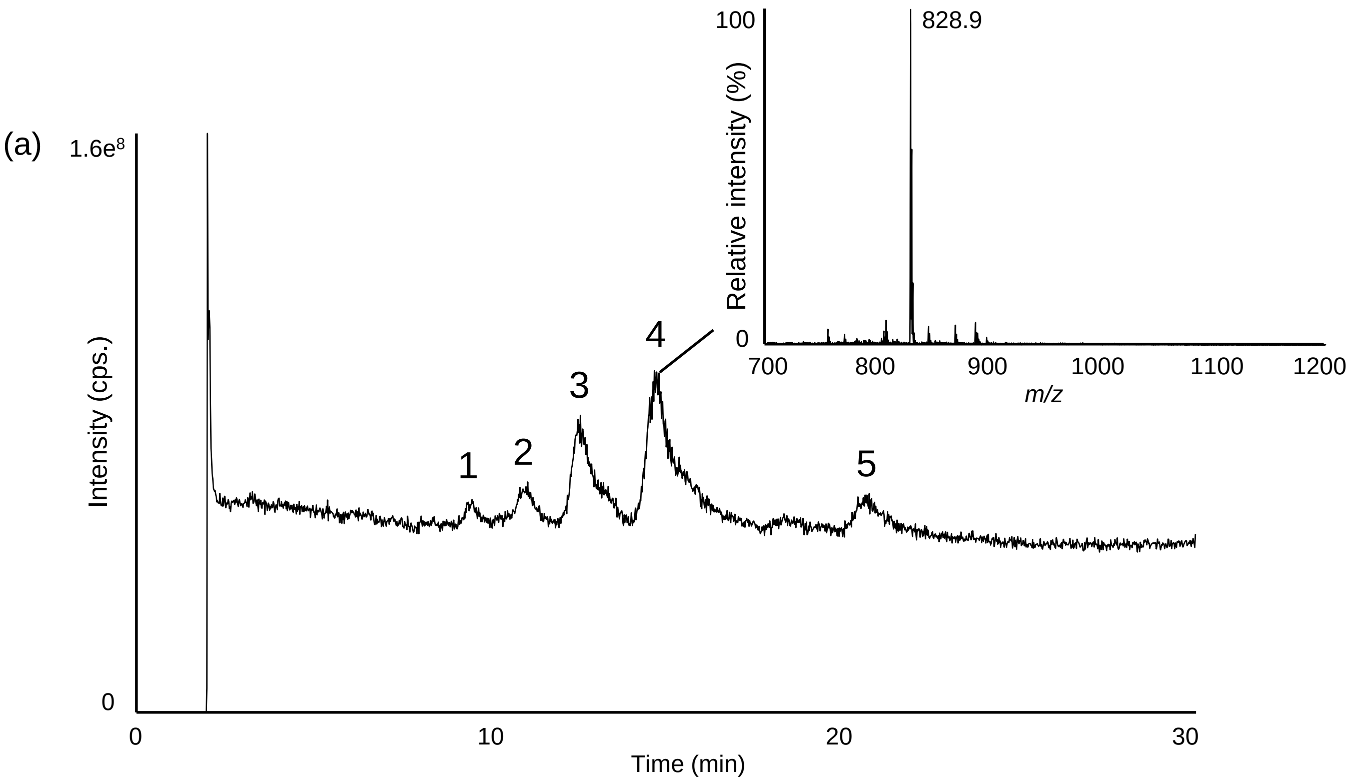

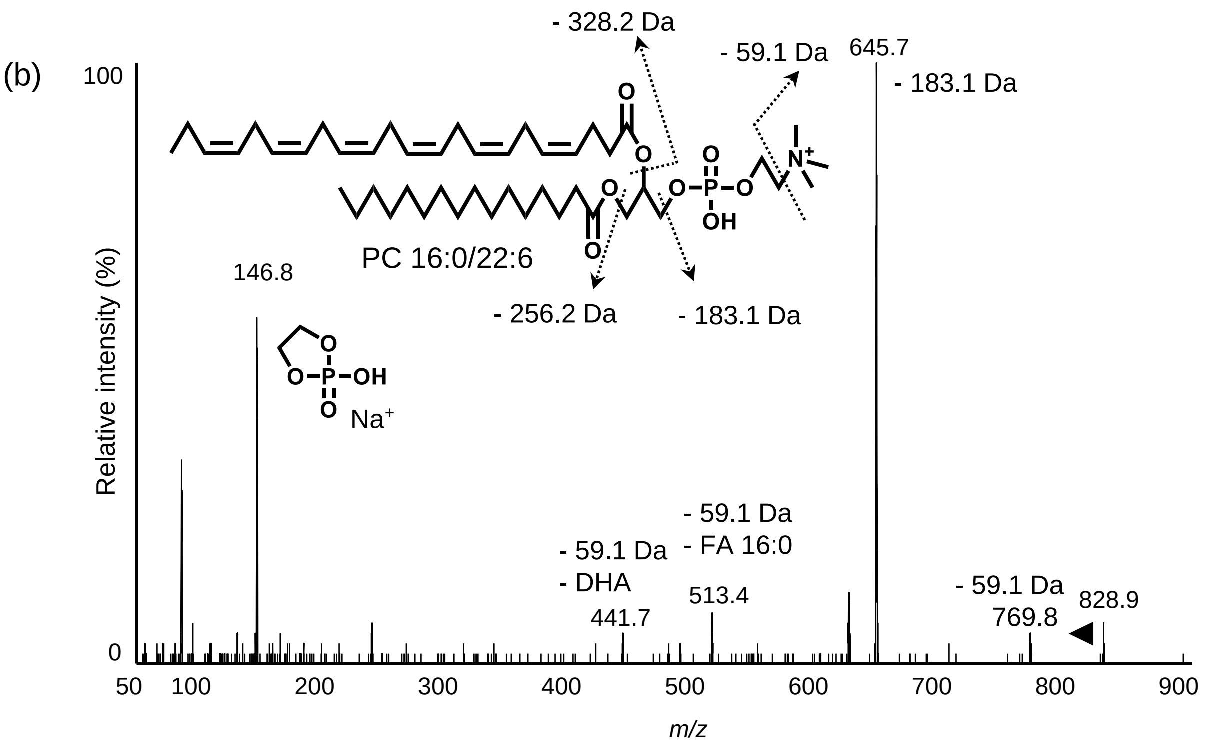

Figure S1 (a) LC-MS chromatogram of phospholipid fraction extracted from mackerel and MS spectrum of the highest peak. Peak 1 and Peak 2 were detected at multiple *m/z* (e.g., Peak 1: *m/z* 874.9, *m/z* 850.9, *m/z* 800.9, and *m/z* 774.9; Peak 2: *m/z* 900.9, *m/z* 877.0, *m/z* 826.9, *m/z* 800.9, and *m/z* 776.9). Peak 3 and Peak 5 mostly consisted of *m/z* 802.9 and *m/z* 783.0, respectively. Besides being lower in abundance, these peaks consisted of multiple PC species and/or did not contain PC-DHA, thus we considered PC 16:0/22:6 (*m/z* 828.9 in Peak 4) would be the most suitable target.
(b) Product ion mass spectrum of *m/z* 828.9 in the phospholipid fraction and the determined PC structure.

Figure S2

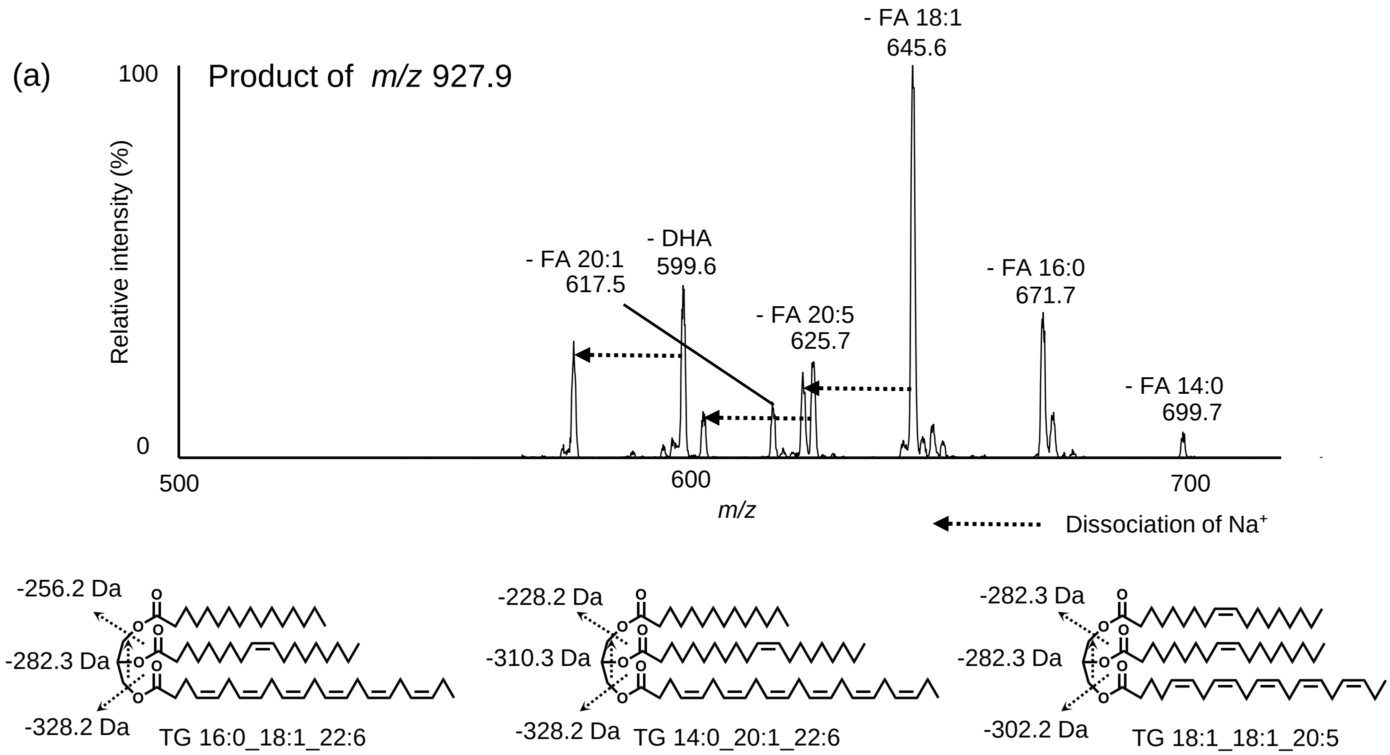

m/z 927.9 – *m/z* 671.7 = 256.2 Da
m/z 927.9 – *m/z* 645.6 = 282.3 Da
m/z 927.9 – *m/z* 599.6 = 328.3 Da

m/z 927.9 – *m/z* 699.7 = 228.2 Da
m/z 927.9 – *m/z* 617.5 = 310.4 Da
m/z 927.9 – *m/z* 599.6 = 328.3 Da

m/z 927.9 – *m/z* 645.6 = 282.3 Da
m/z 927.9 – *m/z* 625.7 = 302.2 Da

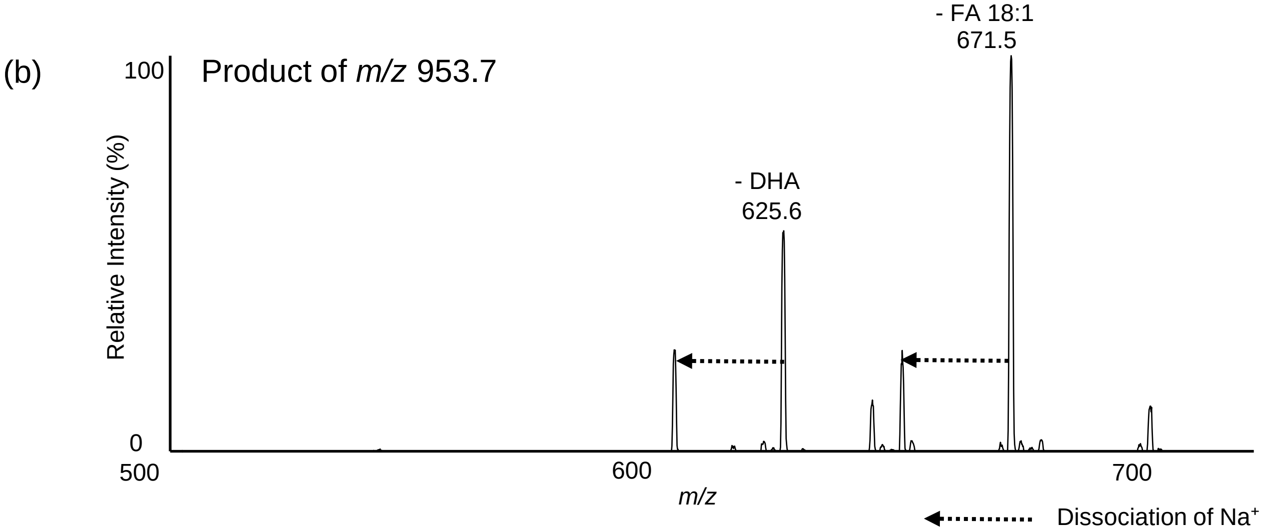

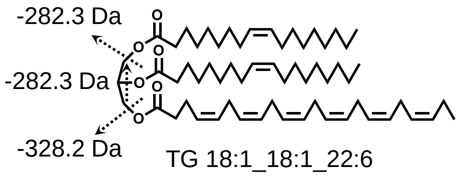

m/z 953.7 – *m/z* 671.5 = 282.2 Da
m/z 953.7 – *m/z* 625.6 = 328.1 Da

Figure S2 (a) Product ion mass spectrum of *m/z* 927.9 in the neutral lipid fraction and the determined TG structures. (b) Product ion mass spectrum of *m/z* 953.7 in the neutral lipid fraction and the determined TG structure.

Figure S3 (a)

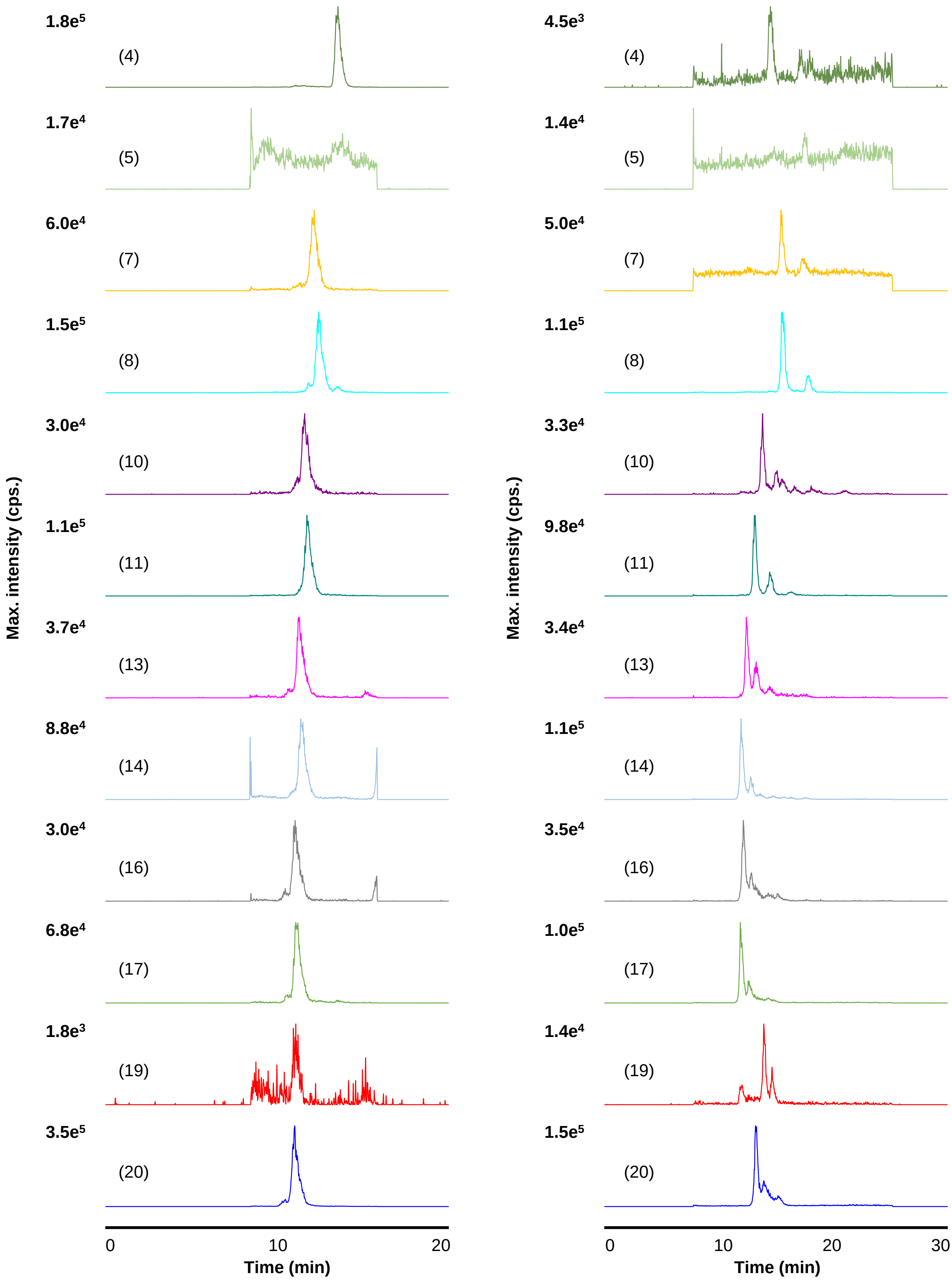

Figure S3 (a) Typical LC-MS/MS chromatograms of PC 16:0/22:6;OOH (left) and TG 18:1_18:1_22:6;OOH (right) in fresh mackerel.

Figure S3 (b)

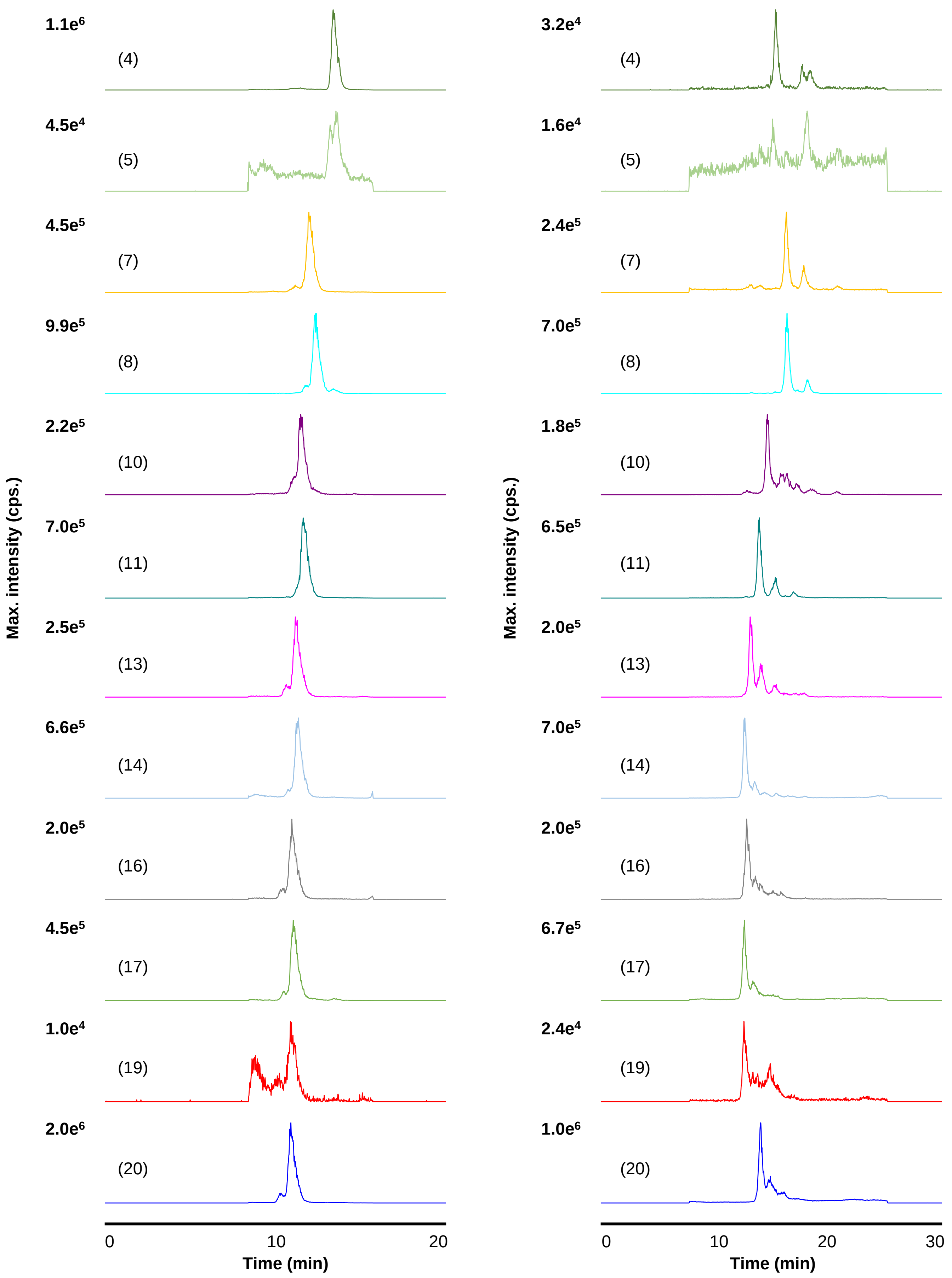

Figure S3 (b) Typical LC-MS/MS chromatograms of PC 16:0/22:6;OOH (left) and TG 18:1_18:1_22:6;OOH (right) in LED-irradiated mackerel (2,000 lx, 3 days).

Figure S3 (c)

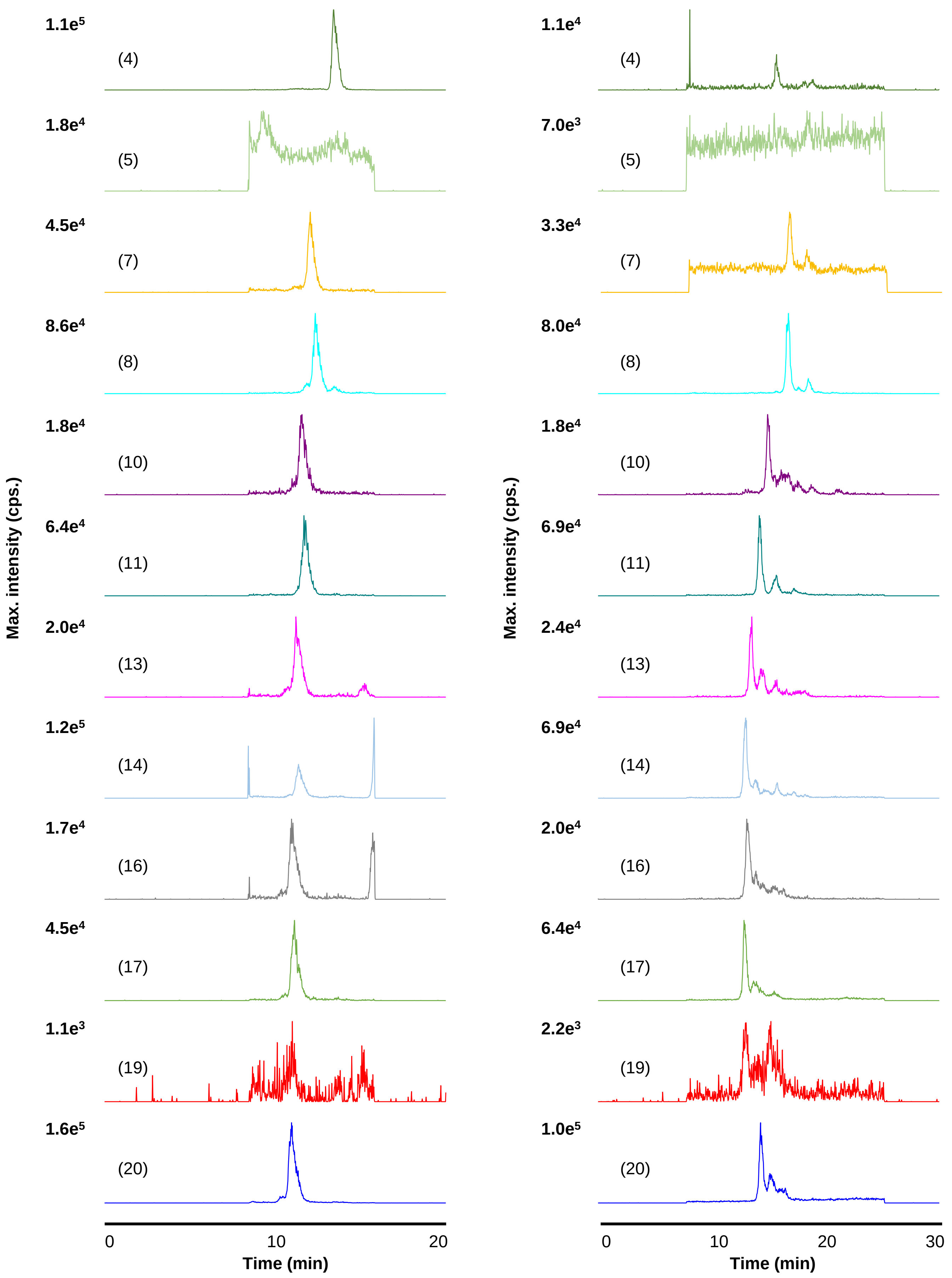

Figure S3 (c) Typical LC-MS/MS chromatograms of PC 16:0/22:6;OOH (left) and TG 18:1_18:1_22:6;OOH (right) in heated mackerel (100ºC, 8 minutes).
